# Does the position of conus medullaris change with increased thoracolumbar kyphosis in ankylosing spondylitis patients?

**DOI:** 10.1097/MD.0000000000005963

**Published:** 2017-02-10

**Authors:** Zhe Qu, Bang-ping Qian, Yong Qiu, Yun-peng Zhang, Jun Hu, Ze-zhang Zhu

**Affiliations:** a Spine Surgery, Drum Tower Hospital of Nanjing University Medical School, Nanjing; b Orthopedic Surgery, the Affiliated Hospital of Xuzhou Medical University, Xuzhou, China.

**Keywords:** ankylosing spondylitis, conus medullaris, magnetic, resonance imaging, thoracolumbar kyphosis

## Abstract

To date, only a few reports described the potential factors influencing the position of conus medullaris. One previous study revealed no significant change of conus locations in patients with idiopathic scoliosis; however, the effect of ankylosing spondylitis (AS)-related thoracolumbar kyphosis on conus position remains unexplored. Therefore, we aimed to investigate the variation of conus medullaris terminations in patients with thoracolumbar kyphosis secondary to AS when compared with normal subjects, and evaluated the relationship between conus positions and the magnitude of kyphosis. In this study, MR images of 96 AS patients with thoracolumbar kyphosis, including 86 males and 10 females with an average of 34.6 years (range, 17–65 years), and 100 age-matched normal controls were reviewed to determine the conus terminations in relation to spinal levels. Sagittal parameters of the AS group measured on radiograph included: global kyphosis (GK), thoracic kyphosis (TK), lumbar lordosis (LL), and thoracolumbar junction (TLJ). Finally, conus tips located at the mean level of the lower 3rd of L1 in both groups, there was no significant difference of the conus distributions between AS and control group (*P* = 0.49). In addition, conus medullaris displayed similar positions in AS patients among various apical region groups (*P* = 0.88), and no significant difference was found when AS population was stratified into GK ranges of 30° (*P* = 0.173). Also, no remarkable correlation of the conus positions with GK (*r* = −0.15, *P* = 0.15), TK (*r* = −0.10, *P* = 0.34), LL (*r* = −0.10, *P* = 0.32), and TLJ (*r* = −0.06, *P* = 0.54) was identified. This study showed the conus terminations displayed a wide range of distributions in AS patients with thoracolumbar kyphosis, which was similar to normal subjects. Moreover, the conus located at a relatively fixed position and would not be affected by the change of kyphosis magnitude, which is an important knowledge that surgeons should acquire in surgical correction of the deformity in these patients.

## 1 Introduction

The termination of conus medullaris with respect to the vertebral levels in normal population was described in various studies.^[1–4]^ Previous reports have documented that the conus reaches to the adult position before 2 years of age, with a range span extended from T12 to L3.^[5–8]^ However, the factors that may influence the conus position after attaining its final level remains largely unknown. As described in some investigations on normal subjects, factors such as age and gender were mentioned to be associated with the location of conus medullaris;^[2,9]^ however, studies evaluating the conus positions in those with neurological abnormalities or spinal deformities are rare. In one report aimed to determine the conus distributions in patients with Chiari I malformation, Tubbs et al^[10]^ detected no remarkable relationship between the conus terminations and the degree of tonsillar ectopia. More recently, Sun et al^[11]^ compared the conus levels in severe idiopathic scoliosis patients with healthy adolescents, they found no significant difference of the conus positions between the 2 groups, and the level of conus medullaris was not correlated with the severity of coronal deformity. Nevertheless, all aforementioned studies gave no answer to the question of whether the conus position would be changed in patients with fixed sagittal deformities.

Ankylosing spondylitis (AS) is a chronic inflammatory disease mainly affects axial skeletons leading to progressive bony fusion of sacroiliac joints and the spine.^[12]^ In advanced stage of the disease, rigid sagittal deformity featured as loss of normal thoracolumbar/lumbar lordosis (LL) or increase of the thoracic kyphosis (TK) with the neck thrust forward may be developed.^[13,14]^ However, as the posterior element of the spine is elongated by the AS-related thoracolumbar kyphosis,^[15]^ whether conus medullaris in these cases terminated at a higher position with corresponding spinal levels is still unknown. Moreover, the effect of kyphotic deformity on conus position is important in surgical decision-making for osteotomy. Therefore, the purpose of present study was 2-fold: 1st, to determine the position of conus in subjects with thoracolumbar kyphosis secondary to AS and compare with normal subjects; and 2nd, to investigate the relationship between conus levels and the severity of thoracolumbar kyphosis.

## 2 Materials and methods

After obtaining approval from the ethical committee (number, 2011052), a retrospective review from January 2007 to December 2014 was carried out of all AS patients underwent 1- or 2-level pedicle subtraction osteotomy (PSO) to correct the secondary thoracolumbar/lumbar kyphosis in our institution, and those with: preoperative magnetic resonance imaging (MRI) of the thoracic and lumbar spine performed, and standing anteroposterior and lateral radiographs of the whole spine available were recruited. Finally, 118 of the 211 initially identified patients had MRI and X-ray for review. Moreover, subjects were excluded if they presented with: concomitant coronal deformity more than 10°; spinal fracture or pseudarthrosis formation; lumbosacral transitional vertebrae; or prior spinal surgery. By these exclusion criteria, 15 cases with pseudarthrosis, 3 with spinal fracture, and 4 with lumbarized S1 vertebrae were excluded. Hence, 96 patients were ultimately enrolled for analysis. They were 86 male and 10 female patients with a mean age of 34.6 years (range, 17–65 years). And none of them showed tethered cord syndrome or neurological deficits. In addition, a total of 100 age-matched (*P* = 0.16) outpatients had lumbar MRI and X-ray for review were selected as the control group, consisting 80 males and 20 females with an average age of 36.6 years (range, 17–57 years). All those in control group were referred for imaging because of low back pain or discomfort, except for some mild degenerative changes, no significant pathologic changes that might influence the position of conus medullaris was found on MRI. In addition, none of those in control group was found to have transitional vertebra on radiograph.

In AS group, the following parameters were measured on standing lateral X-ray of the entire spine to evaluate the magnitude of kyphosis: Global kyphosis (GK),^[13]^ defined as the Cobb angle between the superior endplate of the maximally tilted upper end vertebra and the inferior endplate of the maximally tilted lower end vertebra; TK,^[16]^ measured from the superior endplate of T5 to the inferior endplate of T12; LL,^[17]^ measured between the superior endplate of L1 and S1; and Thoracolumbar junction (TLJ),^[18]^ referred to the Cobb angle from the superior endplate of T10 to the inferior endplate of L2. All of these measurements were defined as positive value with kyphosis and negative value with lordosis. Moreover, the apex of thoracolumbar kyphosis was also recorded.

### 2.1 MRI measurements

**Figure 1:**
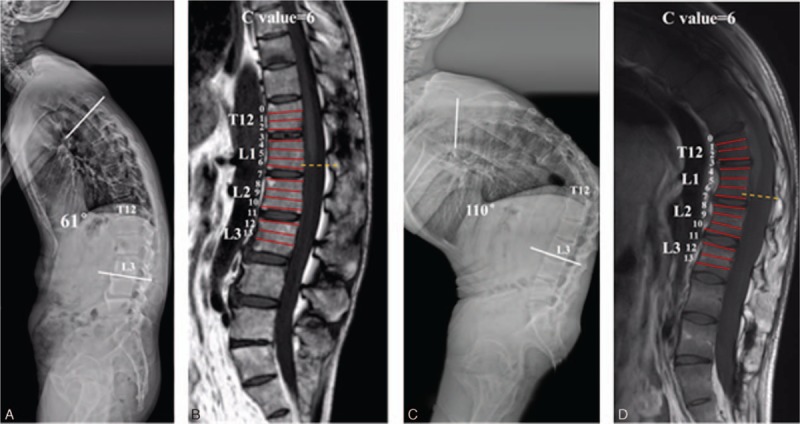
A 53-year-old male AS patient with GK of 61° on lateral standing radiograph (A). Sagittal T1-weighted MR image demonstrated the measurement of the level of conus medullaris, and the conus termination was ascribed to the corresponding 3rd of the vertebral body by drawing a perpendicular line to the long axis of the conus medullaris (B). C value of this case was recorded as 6. In another 24-year-old male patient presented with more severe thoracolumbar kyphosis of 58° (C), the conus position also terminated at the lower 3rd of L1 (C value = 6) (D). AS = ankylosing spondylitis, GK = global kyphosis, MR = magnetic resonance.

T1-weighted sagittal MR images of the lumbar spine showed from T11 to S1 were reviewed in both AS and control groups. Examinations were performed on a 1.5T Symphony system (Philips Medical Systems, Best, The Netherlands) with the patient in the supine position, and the scan parameters of TR 5000 ms, TE 120 ms, FOV 250 mm, Matrix size 250 × 360, and 4-mm thicknesses with a 5-mm gap between the slices were applied. The location of conus medullaris was identified in relation to the adjacent vertebra from picture archiving and communication system (PACS, version 11.4; Philips, Hamburg, Germany), according to the standard method of Saifuddin et al^[4]^ (Fig. 1). In the 1st step, 3 midsagittal images were used to determine the tip of the conus medullaris, which was defined as the most caudal point of the cord that could be visualized. Then, a line perpendicular to the long axis of the cord was drawn to locate the conus level relative to the corresponding vertebra. From T12 to L3, each vertebral/disc unit was divided into 4 segments, including 3 equal vertebral body portions (upper [U], middle [M], and lower [L] thirds) and a separate intervertebral disc region. To facilitate statistical analysis, each segment was assigned a numerical value (C value) ranging from 0 (upper 3rd of T12) to 13 (middle 3rd of L3), and the last rib-bearing segment was counted as T12.^[1]^ Therefore, the position of conus medullaris could be represented by C value.

### 2.2 Statistical analysis

SPSS software version 19.0 for Windows (SPSS Inc., Chicago, IL) was applied for statistical analysis. The distribution of conus positions as C value was calculated and compared between AS and control group using nonparametric Mann–Whitney *U* test. Moreover, AS patients were divided into 3 subgroups based on different apical regions (at T9-T11/12 disc, T12-L1/2 disc, and L2-L4), and 3 groups according to the kyphosis magnitude (GK < 60°, 60° ≤ GK ≤ 90°, and GK > 90°),^[11]^ χ^2^ test and one-way analysis of variance (ANOVA) were performed to evaluate the difference of conus terminations among the subgroups. Spearman correlation coefficient was used to describe the relation between conus positions (C value) and the sagittal parameters. All measurements were performed 3 times by an independent orthopedic resident, and the average values were calculated. Results were considered significance at *P* < 0.05. Moreover, the G∗power software (version 3.1, Germany) for windows was used for the power analysis calculation.

## 3 Results

### 3.1 Conus medullaris termination in both as and control groups

**Figure 2:**
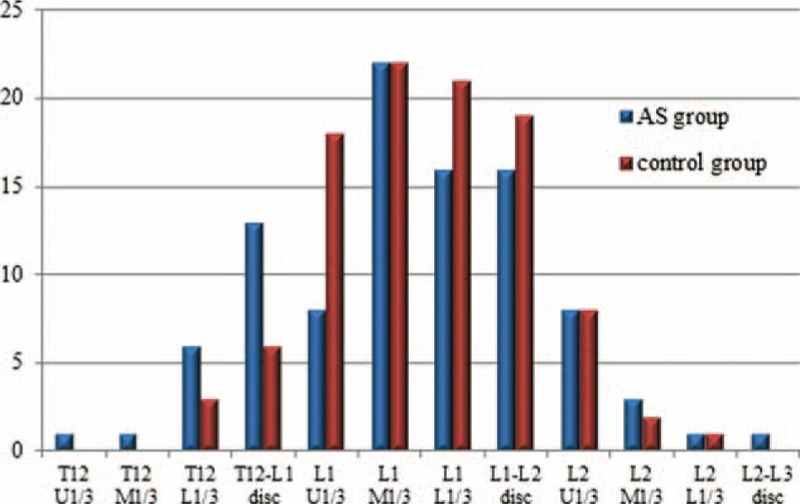
Frequency distribution of the conus position in ankylosing spondylitis (AS) and control groups.

The frequency distributions of C value and conus medullaris level in both AS and control groups were provided in Table 1 and represented graphically in Fig. 2. In AS patients, the conus medullaris terminated from the upper 3rd of T12 (C value = 0) to the L2/3 disc space (C value = 11). And the mean of the C value in AS group was 5.4, which equates to the lower 3rd of L1. Similarly, the mean segment in control group occurred at the same level with an average C value of 5.6; while, the range span of conus positions in normal subjects extended from the lower 3rd of T12 (C value = 2) to the lower 3rd to L2 (C value = 10). According to the nonparametric Mann–Whitney *U* test, no significant difference in the level of conus medullaris between the 2 groups was identified (U = 4528.5, Z = −0.7, *P* = 0.49). The power analysis was conducted using the G∗power software to test the efficacy of the sample size of our study. Finally, a power of 81.0% was achieved, indicating that the statistical analysis was powerful enough to support our findings.

**Table 1:**
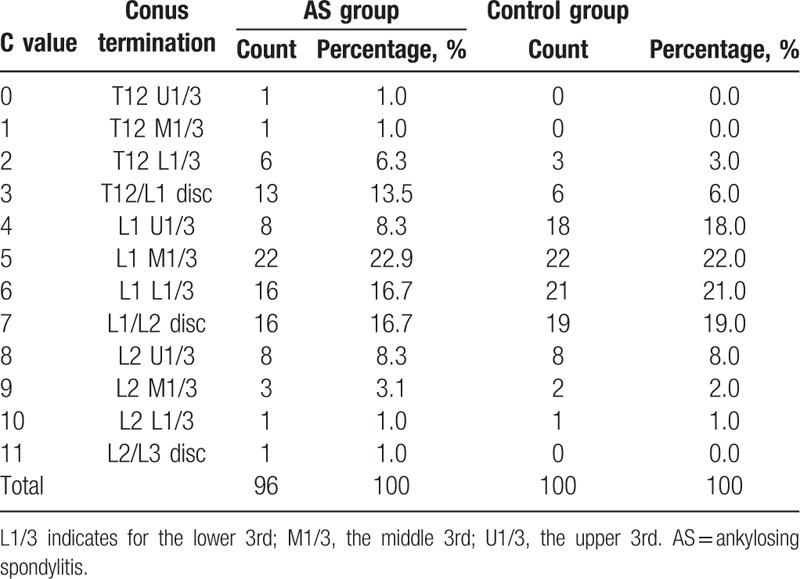
Frequency distribution of the conus medullaris termination in AS and control groups.

### 3.2 Conus positions with reference to different apical regions

In assessing the conus distributions in AS patients of different apical regions, subgroups of the apical vertebrae/discs within the level of T9-T11/12 disc, T12-L1/2 disc, and L2-L4 were divided, and the average C values in these groups were 5.2, 5.3, and 5.5, respectively. Based on the mean (μ = 5.6) and standard deviation (s = 1.6) of the C values in the control group, the μ − s and μ + s were counted as 4 and 7, respectively. Therefore, the conus termination was stratified into 3 subgroups of less than 4, 4 to 7, and greater than 7. The distribution of conus positions in AS patients with various apical regions according to subgroups of C values was shown in Table 2. And no significant difference of conus distributions was observed (the Fisher exact test, *P* = 0.65). In addition, one-way ANOVA comparing mean conus terminations as a function of apical region revealed similar conus levels among the 3 groups (*P* = 0.88).

**Table 2:**
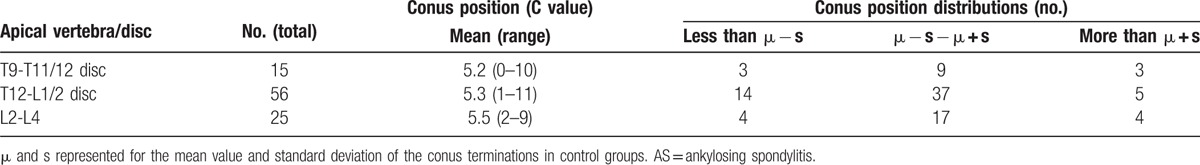
Distribution of conus positions in AS patients of various apical regions.

### 3.3 Correlations between conus terminations and sagittal parameters

The average GK, TK, LL, and TLJ in the study population were 71.3° ± 19.8° (range, 32°–132°), 43.7° ± 17.0° (range, 5°–97°), −0.1° ± 16.6° (range, −51°–44°), and 33.7° ± 13.7° (range, 11°–74°), respectively. To evaluate the effect of curve severity on conus terminations, 3 subgroups with the range of GK < 60°, 60° ≤ GK ≤ 90°, and GK > 90° were made as displayed in Table 3. The one-way ANOVA confirmed that conus distributions did not differ significantly among these groups (*P* = 0.173) with an average C value of 6.0, 5.1, and 5.3, respectively. From the Spearman correlation coefficient (Table 4), there was no significant correlation between the level of conus medullaris and GK (*r* = −0.15, *P* = 0.15), TK (*r* = −0.10, *P* = 0.34), LL (*r* = −0.10, *P* = 0.32), as well as TLJ (*r* = −0.06, *P* = 0.54). Considering our data, it is reasonable to conclude that the level of conus medullaris might not be influenced by the severity of kyphosis (Fig. 2).

**Table 3:**
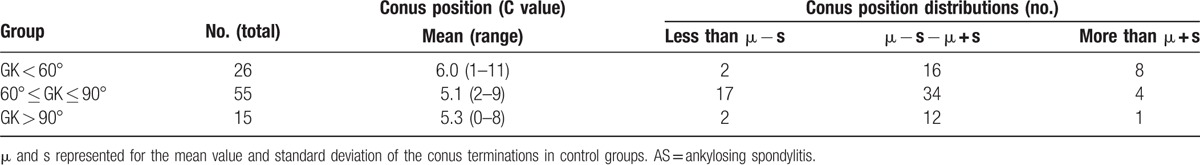
Distribution of conus positions in AS patients of various global kyphosis.

**Table 4:**
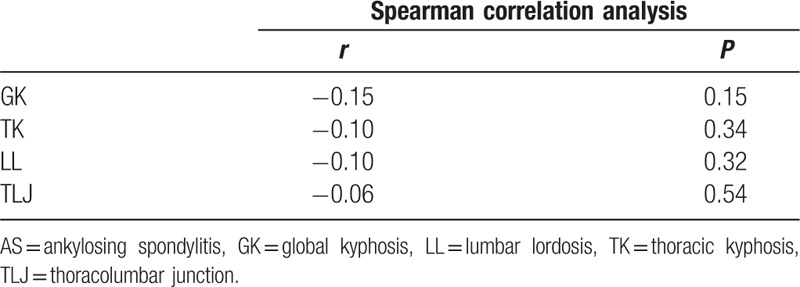
Results of the spearman correlation analysis between conus terminations and the sagittal parameters in AS group.

## 4 Discussion

Due to the asymmetric growth of the spinal cord relative to vertebral column during normal fetal development, the position of conus medullaris ascends gradually from the sacral region to its final level with the increase of gestational age.^[5]^ In an early postmortem study of 252 infants, Barson^[6]^ found that the conus displayed a rapid ascent to L4 vertebra before 17 weeks’ gestation, and then reached an adult level between L1 and L3 with a much slower rate. To evaluate the conus position in a living child population, Wilson and Prince^[7]^ performed an MRI study with 184 children aged from newborn to 20 years, and concluded that not throughout childhood but rather sometimes during the 1st few months did the conus located at the final level. In the adult population, Saifuddin et al^[4]^ first conducted a MRI investigation of 504 subjects, they noted that the mean conus position was the lower 3rd of L1 with the cord span between middle 3rd of T12 to the upper 3rd of L3. More recently, Demiryurek et al^[3]^ determined the level of conus tip varied from T11/12 disc space to the upper 3rd of L3, which was most commonly terminated at the T12/L1 intervertebral disc level. However, whether the conus medullaris is at a fixed position or it would be changed under special conditions after reaching the adult level remains uncertain.

Thomson^[19]^ analyzed the conus positions of 198 cadavers and found the cord tended to be longer relative to the vertebrae in female; while, MRI studies could not support this tendency and showed no significant difference of the conus levels between men and women.^[4,11]^ In assessing the effect of age on conus levels, Demiryurek et al^[3]^ reported no remarkable change of the conus positions among various age groups, which was inconsistent with the study of Soleiman et al^[2]^ that older people had lower positions of conus medullaris compared with younger one. However, all above-mentioned studies were carried out in normal subjects. According to one report regarding patients with Chiari I malformation, the conus position was not affected by the existence of tonsillar ectopia.^[10]^ Besides neurological abnormalities, one study on conus levels with anatomical variations of the spine revealed no remarkable change of the conus positions with the presence of transitional vertebra when counting down from the 1st nonrib-bearing segment as L1.^[1]^ Another study compared conus positions between idiopathic scoliosis patients and normal controls, no significant difference of the conus distributions between the 2 groups was identified, and the conus terminations was not associated with the scoliosis magnitude.^[11]^ Although, because of the elongation of the posterior element of the spine in patients with rigid kyphosis such as AS, whether conus positions would be influenced by the severity of thoracolumbar kyphosis in these patients remains unknown.

To the best of our knowledge, this MRI investigation is the first to have enough power in detecting the influence of thoracolumbar kyphosis on conus positions in advanced stage of AS. The results of our study indicated that conus medullaris lies at an average position of the lower 3rd of L1 in both AS and control groups, which was consist with previous studies of normal subjects.^[4,11]^ Moreover, we found a wide range span of conus positions in AS group extended from the upper 3rd of T12 to L2/3 disc level, which was broader than that of normal controls from the lower 3rd of T12 to L2. However, the comparison of conus distributions between AS and control groups displayed no significant difference. Additionally, there was no obvious change of conus positions in AS patients with different apical regions of the thoracolumbar kyphosis. Given all cases included in current study showed no neurological deficit, we believe that normal conus position in AS patients should be regarded as a range but rather one single level.^[20]^

With respect to the relationship between conus positions and kyphosis magnitude, the conus distributions were similar among the 3 subgroups with the study population stratified into GK ranges of 30°. Similarly, the correlation analysis also revealed no remarkable association between conus terminations and sagittal parameters, including TK, GK, LL, and TLJ. Considering our data, the level of conus tips was invariable in relation to the increasing thoracolumbar kyphosis. In a previous anatomical study focused on the cord movement with the change of posture, no ascent of conus tips during flexion of the spine was observed.^[21]^ The present study further confirmed that the conus termination would not ascend relative to the spinal level in those with fixed flexion deformity of the thoracolumbar spine. Given all these findings, the conus position is an anatomic parameter determined by fetal development, which is hardly affected by postnatal factors. Therefore, the spinal cord would not be strained or tethered in severe thoracolumbar kyphosis patients. Even though no study reported tethered cord syndrome complicated AS, cases with the co-occurrence of tethered spinal cord and congenital kyphosis were not uncommon^[22–24]^; however, which of the 2 entities antedated the other is unsure. From our point of view, the tethered spinal cord is not a secondary change to spinal deformity but rather an independent pathological change.

Knowing more about the conus terminations in patients with kyphotic deformity is beneficial in the surgical correction of AS-related thoracolumbar kyphosis. With the progress of spinal deformity, patients’ ability of lie down, stand or sit in an upright posture, and look straight ahead would be impaired in severe cases, inducing a great decline in their quality of life.^[25,26]^ In these cases, posterior thoracolumbar/lumbar osteotomy is necessary to restore the sagittal alignment. At present, pedicle subtraction osteotomy (PSO) is the most commonly used 3 column osteotomy technique, which resected articular processes, pedicles, in combination with the posterior wedge of vertebral body to get a high-grade osteotomy and great reconstruction of sagittal alignment.^[27–30]^ PSO is technically demanding and usually performed through L1, L2, or L3.^[31–33]^ And the spinal cord injury is an important concern during this kind of procedure.^[34]^ One recent systematic review documented that the rate of neurological complications was 5% for PSO in AS patients.^[35]^ As the conus medullaris will not ascend relative to the corresponding vertebrae with gradual elongation of the posterior element in AS-related thoracolumbar kyphosis,^[36]^ the risk of medullary cone injury inevitably exists when PSO is performed at L1. Besides, owing to the wide range of conus distributions in AS patients, which could be as low as L2/3 disc space, PSO through L2 and L3 still carries the potential risk of spinal cord injuries. Accordingly, preoperative MRI examination is of great value, and PSO performed caudal to the vertebra that conus tips paralleled would be safer. In addition, no significant influence of kyphosis magnitude on conus positions indicated that the restoration of sagittal alignment in AS patients might not lead to the migration of conus medullaris, and traction of the nerve root or cauda equina would not occur after corrective surgery. However, further studies comparing the conus levels between pre- and postoperation are needed to confirm the effect of corrective surgery on conus positions.

One potential limitation of the present study should be emphasized. The positions of conus medullaris were recorded relative to the corresponding vertebral levels, rather than height-corrected distance measured according to one anatomic landmarker. The measurement adopted in the current study enables economic estimate of conus level to the adjacent vertebra; however, the decreased accuracy may bring potential negative effects on statistical analysis.

In conclusion, the current study investigated the position of conus medullaris in AS-related thoracolumbar kyphosis patients for the first time. We found that the conus occupied the similar distribution in AS group to age-matched normal control; and no significant difference of conus levels among subgroups with various apical regions was detected. Furthermore, GK magnitude did not exert significant effect on conus positions; and there was no remarkable correlation between sagittal parameters and conus positions. The results of our study indicated that the position of conus medullaris relative to the corresponding vertebrae would not be changed with the increasing magnitude of AS-related thoracolumbar kyphosis, an important knowledge that surgeons should acquire in surgical correction of the deformity in these patients.
